# Comparison Between the Impact of Vasopressors and Goal-Directed Fluid Therapy on the Management of Free Flap Reconstruction of Head and Neck and Monitoring in ICU

**DOI:** 10.7759/cureus.12108

**Published:** 2020-12-16

**Authors:** Ghiath Al Saied, Homood M Almutairi, Yousef Alharbi, Muhannad Almohanna, Abdulrahman Almutairi

**Affiliations:** 1 Internal Medicine - Critical Care, King Fahad Medical City, Riyadh, SAU; 2 Otolaryngology, Unaizah College of Medicine, Qassim University, Buraydah, SAU; 3 Medicine, Unaizah College of Medicine, Qassim University, Buraydah, SAU

**Keywords:** free flap reconstruction of head and neck, vasopressors, goal-directed fluid therapy

## Abstract

Head and neck reconstructions are often accompanied with complex long surgical procedures. Free flap tissue transfer is a standard reconstruction method that reestablishes severe tissue defects after resection due to trauma or cancer. Imbalanced fluid resuscitation can extremely harm the outcome of the flap either due to hypoperfusion or edema. Flap-related postoperative complications mainly flap failure necessitates the administration of a large amount of intravenous fluids perioperatively especially with lengthy operative time. Therefore, vasopressors may be used to preserve hemodynamic stability without excessive fluids use. Nevertheless, these vasopressors have long been disfavored as they may provoke anastomosis vasoconstriction leading to graft hypoperfusion and finally flap failure. However, according to recent guidelines, they are now well-thought to be safe. Of note, inotropes have been confirmed to increase blood flow in the anastomosis hence they can replace vasoconstrictors. Recently, goal-directed fluid therapy (GDFT) has been proven to be excellent in high-risk head and neck free tissue transfer surgery as it decreases prolonged intensive care unit (ICU) admission hospitalization and complication rate. Today, GDFT is highly suggested as one of the enhanced recoveries after surgery protocols for major head and neck free flap reconstruction surgery.

## Introduction and background

Free flap reconstruction of head and neck involves the microvascular heterotopic free tissue transfer including re-implantation of its blood supply [1]. This technique aims to restore function and cosmetic appearance of head and neck open wounds resulting from trauma or burns, and tumor excision with success survival rates exceeding 95% [2].

Increased risk of complications and higher cardiovascular morbidity and mortality frequently associate head and neck free flap reconstruction. Patients undergoing such procedures usually have low cardiovascular reserves that limit satisfactory organ perfusion with loss of function and even failure [3].

Therefore, ICU close monitoring for the first 24-48 hours post-operatively of the patient and fresh flap, anticoagulation, and fluid resuscitation is essential for the early prevention of complications [4].

Anticoagulants are used in preventing venous thromboembolism in high-risk patients immediately post-operatively especially when a patient’s Caprini risk score is ≥ 8 while they should be stopped 24-48 hours post-operatively if the score is < 8. Caprini score is one of the further most valid in plastic head and neck reconstructive surgery [5]. Numerous anticoagulants such as aspirin, low molecular weight dextran, unfractionated heparin, prostaglandin-E1 are usually used. However, unfractionated heparin was associated with a high number of patients requiring revision surgery of the free flap’s anastomosis [6].

In order to optimize cardiac function and regional blood flow to the flap after adequate fluid delivery, vasopressors as epinephrine, norepinephrine, dobutamine, and dopexamine are usually useful [7].

Vasopressors are often required for patients undergoing general anesthesia to maintain mean arterial blood pressure (MAP) because volatile anesthetic agents can decrease systemic vascular resistance (SVR) in addition to hypothermia as well as blood and fluid losses. Vasopressors are also required to avoid induced hypotension from opioid analgesics, long operative times, and other related medical co-morbidities [7,8].

Recent review of vasopressor use showed a more than 80% lower rate of flap failure in patients [9]. Administration methods include both intravenous bolus and continuous infusion [8,9]. 

A continuously infused low-dose vasopressor drip in combination with the use of neuromuscular blocker to reduce the dose of systemic anesthesia can stabilize MAP and improve flap perfusion after anastomosis [10]. Bolus intravenous administration of vasopressors usually causes wide fluctuations in MAP producing unfavorable effect on the peripheral blood flow needed to perfuse the flap with long-lasting negative effects for its outcome. Therefore, clear coordination of the vasopressor timing, dosage, and agent must be implemented [11].

The intraoperative vasopressor of choice in maintaining normal blood pressure and flap blood flow in hypotensive patients is ephedrine, phenylephrine and norepinephrine [5-7]. Even though there is controversy regarding the use of intraoperative intravenous vasopressors as they induce vasoconstriction, thereby increasing the risk of postoperative thrombosis, decreased flap perfusion, and subsequent flap failure [12] with 70% of the surgeons prohibiting their intraoperative use [11-13]. Notably, these suppositions are chiefly grounded on experimental animal models with contradictory results [12,13]. Nevertheless, recent studies reported that intraoperative vasopressor use in free flap reconstruction did not alter their outcomes neither flap complications nor survival [7-10].

Notably, it was corroborated that there is no relationship between the timing of vasopressor administration either before, during, or after free flap reconstruction and postoperative failure rates [7-10]. On the other hand, no association was observed between the total dose of administered vasopressor and flap failure rates [9].

It has been hypothesized that local catecholamines are released due to sympathetic fibers activation during tissue dissection and free flap harvest [13]. Once the local catecholamines stream is washed-out, the acute hyperadrenergic stage is followed by a non-adrenergic stage with probable increase in collateral blood flow, and then by an increased adrenergic phase due to loss of moderating autonomic contribution in a delayed manner ranging from 48 hours to two weeks [14]. Therefore, the intraoperative use of vasopressors may actually increase flap perfusion due to enhanced overall mean arterial pressure without significant harm from sympathectomy [7,8].

There is an ongoing controversy on whether vasopressors have diverse effects on outcomes of bony versus soft tissue free flap reconstruction [11]. As corroborated in animal studies, bony free flaps typically involve longer procedures time causing increased flap ischemia and often depend on blood supply from periosteal perforators with a higher degree of ischemia relative to vasopressor administration, in contrast to soft tissue free flaps [15].

## Review

Physiological effects of vasopressors

The homeostasis of the body is mainly controlled by the autonomic nervous system, including parasympathetic and sympathetic components. The adrenergic α- and β-receptors are responsible for the vascular tone and cardiovascular function. The α-1 agonists help smooth muscle contraction, resulting in increased systemic vascular resistance and MAP [13]. On the contrary, α-2 agonists cause smooth muscle relaxation and contribute to platelet aggregation through the activation of α-2 receptors on platelets. The positive inotropic and chronotropic effects of β-1 agonists mainly increase cardiac output, while β-2 agonists cause smooth muscle relaxation in the lungs [16].

Ephedrine has lesser effects on peripheral vasoconstriction as it is an indirect sympathetic agonist with primarily strong β-1 and β-2 effects and weak α-1 stimulation. Hypotension is considered when the MAP became lower than 60 mmHg as the arterial blood pressure during anastomosis should be equal to or more than 70 mmHg.

The significant principle of optimizing cardiac performance to enhance free flap perfusion is the goal of fluid management. Based on the current evidence, perioperative goal-directed therapy can improve postoperative outcome of intermediate-to-high-risk surgical patients. It seems to be associated with decreased postoperative length of stay, complications and possibly even mortality. Fluid resuscitation has been chosen for hemodynamic support after free flap ischemia from systemic vasopressors [17].

Significant amounts of intraoperative intravenous fluids are administrated to avoid intraoperative vasopressor use. Fluid resuscitation always carries the risk of both limited and excessive fluid delivery resulting in flap failure with end-organ dysfunction [18].

Colloids should be used cautiously in free flap patients. Patients should be kept hydrated for the sake of improved flap survival. Crystalloid fluid resuscitation is used to ensure adequate flap and renal perfusion with a goal urine output of 0.5-1.0 mL/kg/hour [19].

Crystalloid volumes should not exceed 130 mL/kg per 24-hour period. The controversy about colloid versus crystalloid solutions in fluid resuscitation has long been discussed in the recent years and ended with the favor of crystalloids. A large meta-analysis by Perel et al. accomplished that colloids are significantly more expensive than crystalloids and some of them might even increase mortality rather than reducing it [20].

Greater total intravenous fluid volume of crystalloids than colloids are needed to meet the same hemodynamic stability [19]. The intravenous fluids (IVF) administration is the first-line treatment that combats acute changes in blood pressure, whereas, their excessive administration may result in flap edema and decreased flap microcirculation via hemodilution-induced hypercoagulability contributing to free flap failure [2,7,18].

Free flaps edema is mainly owed to flap denervation that leads to absent lymphatic drainage pathways and poor interstitial fluid reabsorption [7]. Furthermore, deleterious flap and patient outcomes may occur secondary to pulmonary edema and associated cardiopulmonary complications resulting from excessive fluid resuscitation [10-18].

On the other hand, increased free flap complications can also result from under-resuscitation due to decreased flap perfusion, deteriorated by further hypotension from anesthetic agents. Ischemia-reperfusion injury can aggravate inflammation with subsequent flap failure [21]. Therefore, the microvascular surgeons and anesthesiologists must be vigilant in the administration of vasopressors and IVF to improve free flap and patient outcomes. Of note, the use of vasopressors may be an advantageous alternative to IVF to regulate systemic perfusion pressure and flap perfusion intraoperative [5].

It is well known that head and neck free flap reconstruction requires long operative and hospitalization times [22]. Goal-directed fluid replacement (equal weight in the immediate pre- and postoperative period) is one of the protocols specific to head and neck surgery that was designed to optimize patients’ cardiovascular performance and thus proposed to reduce surgical morbidities and reduce the length of stay by utilizing a multimodal and multidisciplinary algorithms [6-21].

Other enhanced recovery protocols include standard perioperative management, especially pre-operative assessment, thermoregulation, hemodynamic and flap monitoring, deep vein thrombosis (DVT) prophylaxis, analgesia and antibiotics guidelines, intensive care unit admission and early mobilization [6].

The goal-directed fluid replacement is mainly dependent on the tissue perfusion physiology. For example, it modulates the adequate oxygen supply to tissues either by its content in blood or the blood flow or depressing organ demands [23]. Although the local tissue blood flow is dependent on systemic blood pressure, it is autoregulated to keep constant blood supply under a wide range of blood pressure. The best example of this hypothesis in case of severe hypoperfusion is the redistribution of blood flow to vital organs (i.e., heart and brain) while gastrointestinal tract, kidneys, skin, etc. may suffer hypoperfusion (occult hypoperfusion). Consequently, controlling cardiac index remains the basis of goal-directed fluid replacement which can be achieved by functional hemodynamic monitoring [22]. Conferring to these data, surgical wound healing is associated with increased tissue oxygen consumption which necessitates an increase of the cardiac output and modulation of systemic vascular resistance in order to pass the perioperative period without organ failure [21].

Goal-directed fluid replacement is possible by modulating any of the parameters affecting stroke volume (SV)-preload, contractility or afterload. For instance, increasing the preload can be achieved using IVF replacement and increasing heart contractility can be attained by inotropes (dobutamine or dopexamine) but with the cost of increased myocardial oxygen consumption. Lastly, afterload is usually moderated to reach adequate perfusion pressures, but with local flow redistribution. Consequently, goal-directed fluid replacement is highly recommended in high-risk surgical patients [24].

Although fluid management has been directed by monitoring standard vital signs such as blood pressure, heart rate and urine output, these endpoints are insensitive to small changes in hemodynamic stability and hypoperfusion [14].

Recently, goal-directed therapy (GDT) is based on the Frank-Starling mechanism which states that the heart contractility increases with greater diastolic filling. When the total circulating blood volume is increased by intravenous fluids administration, cardiac output is thus increased. On the other hand, if hypervolemia occurs, this cardiac output augmentation will reach a plateau, then decreases [21].

In GDT, the intravenous fluids, vasopressors and inotropic administration are guided by instantaneous measurements of stroke volume (SV) and stroke volume variation (SVV) in one respiratory cycle to optimize cardiac output (CO). If hypovolemia occurs, the SVV will be exaggerated, with more response to fluid administration and better hemodynamic stability [22]. In order to perform GDT, the FloTrac/Vigileo system (Edwards Lifesciences LCC, Irvine, CA) is used for monitoring hemodynamic stability. Vigileo monitor continuously analyses the arterial pressure waveform to calculate SV, SVV, CO and cardiac index (CI; CO / total body surface area) through a connected transducer in a radial arterial line [25,26].

To the best of our knowledge, the SVV-guided hemodynamic treatment keeping SVV ≤ 13% improves oxygen delivery, reduces intravenous fluids perioperatively, as well as medical complications and hospital stay compared to conventional fluid management in head and neck patients [27,28].

Predictors of flap failure

Flap failures comprise pre-operative patient characteristics as well as peri- and post-operative factors. Pre-operative aspects including medical status (cardiovascular disease, stroke, asthma, diabetes, chronic obstructive pulmonary disease and renal disease), age, body mass index, smoking, previous blood transfusions, comorbid conditions, chemotherapy and radiation therapy are paramount determinants of outcome [29].

Perioperative variables were collected from intraoperative time, number of flaps, and type of reconstruction, CO monitor, volume and type of administered intravenous fluid, blood transfusion, blood loss, urine output, and administered vasoconstrictors or inotropes [22].

Postoperative variables are the duration of ICU hospitalization, 30-day flap survival and flap-related complications including flap dehiscence, fistula, donor site complications, need for reoperation due to flap failure [14-30].

The major features contributing to free flap failure involve mainly the condition of the tissue, mechanical compression, as well as fibrin dominant venous and platelet dominant arterial thrombosis. These facts indicate using antiplatelet agents or heparin to avoid arterial and venous thrombosis respectively. Surgical manipulations contribute mainly to tissue condition. Outside the native excellence of the flap, rough management of the tissues at the site of injury leads to deteriorating edema and inflammation with associated hypercoagulability [14].

Previous animal studies that examined vasopressor administration in free flaps have reported that flaps were more subtle to vasopressors once detached from the innate nervous structure of the original site of donor tissue aggravating flap failure. Mechanical compression varies according to patient movement, surgical tailoring, or hematoma following uncontrolled and recurrent bleeding [31].

Monitoring of head and neck free flaps

Moreover, it was proven that near-infrared spectroscopy (NIRS) can continuously measure free flap tissue oxygenation and perfusion hence predicting microvascular thrombosis in free flaps even before clinical signs, allowing great opportunity for flap rescue [32].

It is a non-invasive well-accepted modality that evaluates flap viability at the bedside directing attention to impending flap failure, allowing prompt surgical intervention. Another modality that evaluates oxygen saturation percent (StO2%) in the tissues beneath the sensor is tissue oximetry that uses two wavelengths of 690 and 830 nm near-infrared light which spread to the tissue, and then collected by four photo detectors located in each sensor. Measurements can be affected by the probe position [32].

NIRS is more advantageous than laser Doppler modality in identifying arterial flow complications as its values are not affected by the patient movement [33]. Also, NIRS has the advantage of light capability to penetrate deeply into large volume of tissues in a non-invasive, cheap, reliable and reproducible manner. However, laser Doppler flowmetry can only observe superficial microcirculatory changes in tissue [34].

Another promising additional parameter is the new Hypotension Probability Indicator that could be advantageous to discover any drop in the MAP, before hypotension occurs [35].

## Conclusions

Recently, GDFT has been proven to be excellent in high-risk head and neck free tissue transfer surgery as it decreases prolonged intensive care unit admission (ICU)hospitalization and complication rate. Today, GDFT is highly suggested as one of the enhanced recovery after surgery protocols for major head and neck free flap reconstruction surgery.
